# Health-related quality of life in children with flexible flatfeet: a cross-sectional study

**DOI:** 10.1007/s11832-014-0621-0

**Published:** 2014-11-08

**Authors:** Alpesh Kothari, Julie Stebbins, Amy Beth Zavatsky, Tim Theologis

**Affiliations:** 1 Nuffield Department of Orthopaedics, Rheumatology and Musculoskeletal Sciences, Nuffield Orthopaedic Centre, University of Oxford, Windmill Road, Oxford, OX3 7HE UK; 2 Oxford Gait Laboratory, Nuffield Orthopaedic Centre, Oxford University Hospitals NHS Trust, Windmill Road, Oxford, OX3 7HE UK; 3 Department of Engineering Science, University of Oxford, Parks Road, Oxford, OX1 3PJ UK

**Keywords:** Pes planovalgus, Flatfeet, Outcome measures, Health-related quality of life

## Abstract

**Purpose:**

The effect of paediatric flexible flatfeet (PFF) on health-related quality of life (HRQOL) has not been investigated. In this prospective cross-sectional study, the HRQOL of children with PFF was compared to those with typically developing feet (TDF) using two validated measures. We hypothesised that reduced HRQOL would be observed in children with PFF. The reliability of parents’ perceptions of their child’s symptoms was also investigated.

**Methods:**

48 children with PFF and 47 with TDF between the ages of 8 and 15 completed The Oxford Ankle Foot Questionnaire for Children (OxAFQ-C) and Pediatric Quality of Life Inventory (PedsQL™ 4.0). Proxy questionnaires were also completed. Reliability of parent and child questionnaire scores was assessed using the intraclass correlation coefficient (ICC) and Student’s *t* test. Differences between HRQOL between PFF and TDF were assessed using the Student’s* t* test.

**Results:**

ICCs overall demonstrated good reliability between parent and child questionnaire domain scores. There was a tendency for parents to overestimate the impairment of the child in the PFF group. PFF children demonstrated clinically significant decreased HRQOL than TDF children. This was most marked in the physical domain scores.

**Conclusion:**

Although parents may overestimate their child’s impairment, children with PFF still have significantly impaired HRQOL when compared to TDF children. The impairment can be as severe, or worse, than published HRQOL for acutely and chronically unwell children. As such, PFF cannot be regarded as just a benign normal variant. The management of PFF should involve consideration of the symptom profile and HRQOL.

**Level of evidence:**

II.

**Electronic supplementary material:**

The online version of this article (doi:10.1007/s11832-014-0621-0) contains supplementary material, which is available to authorized users.

## Background

Flexible flatfeet in children [paediatric flexible flatfeet (PFF)] are common, with a prevalence between 2.7 and 18.1 % [1, 2]. It is thought to be the most frequent reason for attendance at paediatric orthopaedic clinics [3]. Most clinicians believe that PFF is a normal variant of foot type and requires no intervention [4]. Others believe that, in a proportion of cases, PFF leads to significant foot and ankle symptoms which do require intervention [5]. The exact percentage of cases that develop symptoms is debated, with estimates varying from 10–60 % [5, 6]. It is also estimated that up to 63 % of children with PFF have functional impairment [6]. Symptoms may relate to early muscle fatigue and foot-and-ankle-complex instability, leading to capsule and/or ligament strain and imbalance. It is hypothesised that this leads to progressive deformity, degenerative arthropathy and problems into adulthood. Excessive hindfoot valgus associated with PFF may also affect the normal biomechanical function of the foot and ankle [7].

There have been numerous studies investigating the differences between PFF and typically developing feet in children (TDF). Differences have been found in a number of dimensions, including the static alignment and geometry of the tarsal bones, plantar pressure patterns, and gait kinematics [8–11]. The description of anatomical and functional differences seen in PFF contributes to our understanding, but few studies have looked at the clinical picture and investigated how these differences relate to symptoms. Amongst the few, Moraleda et al. [8] correlated two radiological measurements with symptomatic flat feet, and Hosl et al. [10] used gait analysis to assess kinematic differences between TDF and symptomatic and asymptomatic PFF (SFF, ASFF). Whilst Hosl et al. [10] found differences between the TDF group and PFF as a whole, they were unable to distinguish between SFF and ASFF.

It remains unclear why some children with PFF might develop symptoms and others have no problems whatsoever. When considering the specific symptoms children with PFF experience, complaints are broad and symptoms vary in severity. The symptom profile is quite heterogeneous, but studies tend not to reflect this and use ‘symptomatic’ or ‘asymptomatic’ as a binary classifier [8, 11, 12]. It is also unclear whether the symptoms caused by PFF are severe enough to warrant intervention, and there has been no comparison between this pathology and other foot and ankle pathologies.

Knowledge of the symptom profile and its effect on the health-related quality of life (HRQOL) of the child is important, as it often guides management. In the context of PFF, no study has assessed the effect of PFF on quality of life of the patient.

A number of health-related quality of life measures have been developed to assess general health status in children. One of the most widely applied generic HRQOL measures used in the paediatric population is the PedsQL™ 4.0 generic core scales [13]. This tool developed by Varni et al. [13] was designed to measure the core dimensions of health as outlined by the World Health Organization as well as school functioning. The PedsQL™ 4.0 has been extensively validated and is proposed to be reliable, valid, responsive and developmentally appropriate. The tool can also be used in healthy populations as well as those with pathology. A more condition-specific HRQOL measure, the Oxford Ankle Foot Questionnaire for children (OxAFQ-C), has been proposed to measure HRQOL in children between the ages of 5–16 with foot and ankle pathology [14]. Both measures comprise a patient questionnaire and a validated, proxy questionnaire to be completed by a parent/guardian. In the case of PFF, it is a widely held belief that parental anxiety caused by PFF far outweighs the severity of symptoms experienced by their child [15]. As it is usually the parent/guardian who accesses the health system on behalf of their child, it is important to assess for consistency and bias between patient and proxy questionnaire scores [15, 16].

In this cross-sectional study, we used HRQOL measures to gain a better understanding of the health-related quality of life in children with PFF. There were two main hypotheses:

1. There would be good reliability and no systematic bias between patient and proxy questionnaire scores in PFF and TDF subject groups.

2. A significantly worse HRQOL would be demonstrated in children with PFF compared with children with TDF.

As the OxAFQ-C is a relatively novel HRQOL measure, domain scores have also been benchmarked by assessing their relationship with PedsQL™ 4.0 domains scores.

## Materials and methods

### Data collection

The study was approved by the local research ethics committee (ref: 12/SC/0334). Informed consent from parents/guardians and assent from child participants was obtained before assessment. For the PFF and TDF groups, inclusion criteria were: aged between 8 and 15 years old with a neutral or flat foot posture. Exclusion criteria were: any neurological, bone or joint disease, any previous lower limb operations, or concurrent use of orthoses. Subjects were either recruited from a hospital clinic or the community. Subjects were not selected on the basis of having symptoms but on the basis of foot posture.

### Foot posture classification

**Fig. 1:**
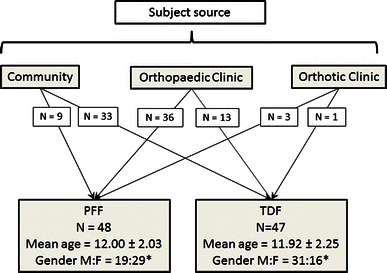
Flow chart demonstrating subject route into study and patient demographics. *N* denotes number of subjects. *Asterisk* highlights statistically significant difference between gender proportions (*p* = 0.01)

Due to concerns about the subjective nature of foot posture classification using visual inspection, an objective technique using a combination of common existing methods was used to classify foot posture. Children all underwent three-dimensional motion analysis, anthropometric measurement, dynamic pedobarography using the Novel EMED-M pressure plate system (Novel, Munich Germany), and simulated weight-bearing MRI. From these assessments, a set of non-correlated foot posture measures for each participant was obtained. Logistic regression of the results of a two-step cluster analysis using these measures was undertaken to classify feet as either PFF or TDF. A detailed description of the cluster analysis method can be found in the supplementary material. The foot posture measurement indices for the PFF and TDF groups were consistent with previous literature [8, 17–20]. Of the 95 children participating in the study, 48 were classified as having PFF and 47 as TDF. Figure 1 summarises the recruitment route into the study, group allocation, and demographic information.

### Measures

The OxAFQ-C questionnaire was developed to assess the reported health status of children aged 5–16 years with foot and ankle problems [14]. It has 15 items, the first 14 of which are used to calculate domain scores (“physical”, “school and play”, and “emotional”). The three domain scores are reported separately as a percentage, where the lower the percentage score, the worse the health status. The final item reflects the concern by many children that they cannot wear the footwear they prefer and is reported as a single item.

The PedsQL™ 4.0 Generic Core Scales tool has been developed through focus groups and cognitive interviews to capture HRQOL in children aged 2–18 (2–5 solely proxy reported) [13]. There are 23 items which encompass physical functioning (8 items), emotional functioning (5 items), social functioning (5 items), and school functioning (5 items). Each item consists of a 5-point Likert scale which is then reverse scored and linearly transformed to a 0–100 scale, so that higher scores indicate better functioning. Domain scores can be reported separately as the mean score of all items in the domain. A total score can also be reported as the mean score of all item scores in the questionnaire.

Each child was instructed to complete both the OxAFQ-C questionnaire and the PedsQL™ 4.0 questionnaire. The parent/guardian of the child was instructed to fill out the proxy questionnaire equivalents. Questionnaires were completed independently by the child and parent/guardian.

### Statistical analysis

Reliability between patient and proxy questionnaires was assessed using the absolute agreement intraclass correlation coefficient (ICC) and comparing mean absolute differences with a paired two-tailed *t* test as per recommendations by Marshall et al. [21] Questionnaire domain scores were compared between subject groups using an unpaired two-tailed Student’s *t* test. Pearson’s *R* was used to assess the correlation between OxAFQ-C and PedsQL™ 4.0 domain scores. Alpha was set at 0.05 to define significance. No correction for multiple comparisons was made for the reasons outlined by Poole. [22]

The sample size was calculated using previously published data by Morris et al. [23] With the published upper limit of minimally important difference of 17 % in the OxAFQ-C physical domain, a maximum standard deviation of 25.2 and a sample size of 47 was required in each group for a 90 % study power. Statistical analysis was undertaken using Stata v.13.0 (Statacorp LP, Texas, USA) and SPSS v 22.0 (IBM, Chicago, IL, USA).

## Results

Comparison of the demographics did not demonstrate any significant difference in mean ages between the groups (*p* = 0.83). There was, however, a significant difference in gender ratios between groups (*p* = 0.01), with the PFF group containing a higher proportion of girls (Fig. 1). Patient questionnaires were fully completed by all subjects. Pairs of patient and proxy questionnaires were obtained for 80 subjects.

### Comparison between patient and proxy questionnaire scores

Indices quantifying agreement between the patient and proxy questionnaires for the combined population are summarised in Table 1. There was fair to excellent agreement between subjects and parents/guardians for the majority of questionnaire domains except for PedsQL™ 4.0 social domain, which only had poor agreement (ICC = 0.38). For the combined PFF and TDF population, there were no significant absolute differences between patient and proxy domain scores for any of the OXAFQ-C questionnaire domains. There was, however, a significant difference between proxy and patients’ PedsQL™ 4.0 physical and emotional domain scores with parents/guardians consistently estimating worse functioning than the child.

**Table 1:** Reliability between patient self-reported and proxy questionnaire scores for all subjects

All subjects	*N*	Mean difference	Sig.	SD of difference	95 (%) confidence interval		ICC
OxAFQ-C physical	80	−0.50	0.979	17.60	−3.97	3.87	0.87
OxAFQ-C School/play	80	−1.60	0.242	12.40	−4.40	1.13	0.84
OxAFQ-C emotional	80	−2.10	0.313	18.60	−6.24	2.02	0.64
OxAFQ-C extra Q	80	−0.94	0.750	26.20	−6.77	4.89	0.81
PedsQL™ 4.0 Physical	80	−3.28	0.010*	11.20	−5.76	−0.81	0.86
PedsQL™ 4.0 Emotional	80	−5.93	0.005*	18.47	−10.01	−1.84	0.38
PedsQL™ 4.0 social	80	−0.09	0.962	16.40	−3.72	3.54	0.38
PedsQL™ 4.0 school	80	0.68	0.711	16.40	−2.96	4.31	0.60

Mean difference is the average of the difference of patient self-reported questionnaire subtracted from the paired proxy questionnaire. Significance of mean diff. is given (sig.), as well as the standard deviation of the difference (SD of difference), and the 95 % confidence intervals of the mean diff *ICC* denotes the intraclass correlation coefficient

*N* denotes sample size

* Statistical significance

Table 2a, b summarise the indices quantifying agreement between the patient and proxy questionnaires when the population was split into PFF and TDF groups. In general, there was a tendency for parents of children in the PFF group to over-estimate the impairment in HRQOL experienced by the children. This reached statistical significance in the PFF group for the PedsQL™ 4.0 Physical domain (*p* = 0.026) and the OxAFQ-C School and Play domain (*p* = 0.041) scores. In the TDF group, negative bias was observed only in the absolute mean score difference in the PedsQL™ 4.0 emotional domain (*p* = 0.002). Better ICCs were observed for OXAFQ-C domain scores than PedsQL™ 4.0 domain scores. In general, patient and proxy questionnaire scores for physical domains demonstrated greater agreement in the groups than the other domains.

**Table 2:** Reliability between patient self-reported and proxy questionnaire scores for PFF (a) and TDF (b) groups

	*N*	Mean difference	Sig.	SD of difference	95 (%) confidence interval		ICC
(a) PFF							
OxAFQ-C physical	44	−3.50	0.230	19.10	−9.31	2.30	0.83
OxAFQ-C school/play	44	−4.68	0.041*	14.77	−9.17	−0.20	0.81
OxAFQ-C emotional	44	−5.82	0.091	22.32	−12.60	0.96	0.57
OxAFQ-C extra Q	44	−5.11	0.262	29.82	−14.18	3.95	0.75
PedsQL™ 4.0 physical	44	−4.09	0.026*	11.90	−7.67	−0.52	0.85
PedsQL™ 4.0 emotional	44	−3.67	0.236	20.46	−9.81	2.48	0.52
PedsQL™ 4.0 social	44	0.29	0.920	19.19	−5.47	6.05	0.27
PedsQL™ 4.0 school	44	2.77	0.277	16.94	−2.31	7.87	0.65
(b) TDF							
OxAFQ-C physical	36	4.17	0.100	14.18	−0.84	9.18	0.78
OxAFQ-C School/play	36	2.08	0.103	7.47	−0.44	4.61	0.84
OxAFQ-C emotional	36	2.43	0.206	11.30	−1.39	6.26	0.60
OxAFQ-C extra Q	36	4.17	0.226	20.27	−2.69	11.02	0.81
PedsQL™ 4.0 physical	36	−2.26	0.200	10.37	−5.76	1.25	0.76
PedsQL™ 4.0 emotional	36	−8.75	0.002*	15.40	−14.00	−3.51	0.46
PedsQL™ 4.0 social	36	−0.55	0.790	12.40	−4.75	3.64	0.52
PedsQL™ 4.0 school	36	−1.94	0.461	15.64	−7.23	3.35	0.38

Abbreviations as per Table 1

* Statistical significance

### Correlation between OxAFQ-C and PedsQL™ 4.0 domain scores

Correlations between the OxAFQ-C domain scores and PedsQL™ 4.0 domain scores were all positive and statistically significant at *p* ≤ 0.01. Thus, higher domain scores in one HRQOL were associated with higher domain scores in the other and vice versa. The correlation between the OxAFQ-C physical domain and the PedsQL™ 4.0 was high (0.80). The corresponding questionnaire’s emotional domains demonstrated moderate correlation (0.44). Both OxAFQ-C emotional and school and play domains also had moderate correlations with the PedsQL™ 4.0 physical domain scores (0.52 and 0.68, respectively). The remainder of the domains with dissimilar constructs had weak correlations of <0.4.

### Comparison of HRQOL measures between PFF and TDF children

PFF children had significantly lower mean scores in all OXAFQ-C questionnaire domains compared to the TDF children. The differences were most marked for the physical domain and the extra question, with PFF children scoring around 20 % less than TDF children. In the emotional and school and play domains, differences between PFF and TDF children were just under 10 %. Results are summarised in Table 3.

**Table 3:** Mean domain scores with standard deviation (SD) for PFF and TDF groups

	PFF		TDF		Mean difference	Sig.
	Mean score	SD	Mean score	SD		
OxAFQ-C physical	63.45	23.78	83.24	20.31	19.79	<0.001*
OxAFQ-C school/play	86.06	18.56	94.54	10.39	8.48	0.007*
OxAFQ-C emotional	85.28	17.59	94.81	11.46	9.52	0.002*
OxAFQ-C extra Q	66.15	32.82	87.23	23.81	21.09	<0.00*
PedsQL™ 4.0 physical	78.06	15.78	91.02	10.02	12.96	<0.001*
PedsQL™ 4.0 emotional	82.08	19.53	88.4	15.78	6.32	0.043*
PedsQL™ 4.0 social	88.02	16.30	91.7	10.44	3.68	0.097
PedsQL™ 4.0 school	78.64	17.97	87.98	12.88	9.33	0.005*

Mean difference as well as significance level (sig) tabulated

* Statistical significance

Analysis of the PedsQL™ 4.0 domain scores showed significantly lower mean scores for the PFF children compared to the TDF children in all but the social domain, which itself was tending towards significance (*p* = 0.097) (Table 3). The differences in mean scores were most marked for the physical domain (12.96 % *p* ≤ 0.001).

## Discussion

The classification and management of PFF continues to be the subject of considerable debate in the paediatric orthopaedic community. Whilst it is evident that there are structural and functional differences between TDF and PFF, how these relate to symptoms is still, in the most part, unclear. One problem in the literature is that children are grouped on the basis of presence or absence of symptoms [8, 11, 12]. Clinicians would agree that children with PFF may present in a variety of ways and that distinction is not black and white. Hosl et al. [10] attempted to identify some differences when they looked at the relative frequency of pain and fatigue in their study population as well as the anatomical location of symptoms. This information was not used in the analysis, and children were all grouped as symptomatic, even though a wide variety of symptom profiles was demonstrated. Using binary groups in such a fashion results in a loss of data resolution [25]. As symptoms tend to be continuously distributed, it makes sense to use a metric that is also more continuous in nature. After all, a child who experiences mild pain when they have been walking for an hour is not the same as a child who struggles to walk short distances because of severe pain or discomfort. The impact of symptoms on HRQOL is important. In this study, we have attempted to gain better insight into HRQOL of PFF children by use of two validated measures, the OxAFQ-C and the PedsQL™ 4.0.

In the first part of the study, we evaluated consistency between patient and proxy questionnaire domain scores. As a combined group there was, generally, good to excellent consistency as measured by the ICC. Consistency was better for the OxAFQ-C than the PedsQL™ 4.0. Significant negative bias was found between patient and proxy scores for the physical and emotional domains of the PedsQL™ 4.0, with parents/guardians suggesting worse functioning than the child reported. Achenbach et al. [25] found that parents were much better at judging more observable external problems in their child, like aggressiveness, than internalised problems like anxiety or sadness. This might explain why, overall, we see better consistency with more observable problems like physical impairment than other less observable issues like emotional or social functioning.

When the subject groups were split into PFF and TDF, an interesting phenomenon was observed. Parents of children with PFF consistently gave scores lower than the children themselves. This was statistically significant for the OxAFQ-C school and play and the PedsQL™ 4.0 physical domains. These findings suggest that parents perceive PFF to have more negative consequences than do the children themselves. Similar findings were also found by Ennett et al. [26] when they demonstrated that mothers of children with juvenile idiopathic arthritis felt that their child was more affected by the disease than did the child. When deciding on a treatment plan for children with PFF, clinicians should be aware of this potential discrepancy between parent and child perceptions. This discrepancy highlights the need to put primary importance on the history from the child and use parental history to corroborate findings.

The main purpose of the study was to compare the HRQOL between PFF and TDF children. It was hypothesised that children with PFF would have worse HRQOL than TDF children. This was demonstrated both with the OxAFQ-C and PedsQL™ 4.0 questionnaires. When using HRQOL measures, statistical differences between subject and group domains scores need to be put in the context of what the minimal important difference (MID) for that domain is. Anything below this difference may not have any clinical importance and, as such, would not be an important finding. Published MID for the PedsQL™ 4.0 for each domain is 4 points [13]. For the OxAFQ-C, the MID varies between domains, being 10–17 % for the physical domain and 7–9 % for the school and play and emotional domains [23]. In this study, the differences between mean questionnaire domain scores for the PFF and TDF groups were all at or above the MID values, except for the PedsQL™ 4.0 social domain scores. The greatest differences between the groups were observed in the physical domain scores for both HRQOL measures.

The OxAFQ-C has been used in two recent studies to quantify burden of disease in other patient groups. Duffy et al. [27] used it to assess HRQOL in children treated for clubfoot. In their sample, children who were surgically treated had mean child reported OxAFQ-C domain scores between 74.0 and 88.4 %. The Ponseti group had mean OxAFQ-C domain scores between 81.9 and 95.7 %. The PFF group in this study had worse child-reported OxAFQ-C scores in all domains compared to the Ponsetti group and in all but the emotional domain compared to the surgically managed group. Kennedy et al. [28] assessed HRQOL related to foot and ankle abnormalities in Hurler’s Syndrome. They reported an average OxAFQ-C score of 44.7 out of 60; however, as an overall combined score has not been validated for this questionnaire, a direct comparison with the children in this study cannot be made.

In this study, the OxAFQ-C was applied to a normal population (TDF) as well as to a pathological group (PFF). The tool itself was developed in a pathological population and, as such, there are no published normative data. As this is the case, the absolute domain scores are harder to interpret. To legitimise use of the tool in this context, we also used the well-established and validated PedsQL™ 4.0, which does have normative data. In comparison with this normative data, it firstly seems that the TDF population scores were better in all domains than the healthy children in the study by Varni et al. [13]. It is unclear why this is the case, although it may be related to the relatively small sample size and specific inclusion criteria in our study. When looking at individual domain scores, as a group, PFF children had worse mean physical domain scores than the published values for acutely unwell children and similar scores to chronically unwell children. This again indicates the as yet unrecognised impact that PFF may have on an individual's HRQOL. For the other domains, the PFF children had similar domain scores to the healthy children. Correlation between PedsQL™ 4.0 and OxAFQ-C domain scores was particularly strong for the physical domain, and thus the impairment seen in the PFF children can be placed in a broader context. Correlations in other domains were less strong, and thus the scores in the other OxAFQ-C domains are less widely applicable. Discrepancies between the two HRQOL measures will be, in part, due to the fact that the PedsQL™ 4.0 is a generic tool, whereas the OxAFQ-C pertains solely to foot and ankle pathology.

In this study, children with PFF, as a group, demonstrated a markedly broad range of questionnaire scores in all domains. The impairment observed varied from essentially normal function in some children with PFF all the way to significant deterioration in quality of life seen in others. These findings highlight the heterogeneity in the clinical presentation of children with PFF, which in turn makes it difficult to define treatment protocols. In an attempt to simplify things, clinicians have further divided PFF children into those who are asymptomatic and those who are symptomatic. The evidence presented in this paper suggests that this is inappropriate, as a binary definition is not sensitive to the variety of impairments that PFF can cause in children. A binary classifier may also be insensitive to clinical change or improvement, i.e., if a symptomatic child does not become asymptomatic, it does not mean that his or her symptoms have not improved. We suggest that when assessing a child with PFF, the clinician should spend time elucidating the nature and extent of symptoms and the effect on quality of life, as symptomatology is not black and white. Use of the OxAFQ-C to achieve this in such a context could be very helpful. If treatment is instituted, repeat administration of the questionnaire will also make it possible to chart improvement. From a research and audit point of view, this is particularly important in the context of PFF, as the benefit of treatment still remains uncertain.

There continues to be controversy about the best way to classify a flat foot. Whilst current classifications concentrate on morphological differences, it appears that this alone is not adequate in identifying those who develop problems. In this study we used a more elaborate classification for PFF, but this still falls short of being an ideal classification method. It is our future aim to identify if there are key structural and functional characteristics which correlate with low HRQOL scores or which may lead to worsening HRQOL scores. We believe that this will lead to a classification which has a better clinical basis and which may guide management.

The main limitation of this study is that there may have been some selection bias in the recruiting of subjects. As the majority of children with PFF were recruited from an orthotic or orthopaedic clinic, this may only represent the tip of the ‘clinical iceberg’, with the asymptomatic majority in the community not represented [29]. An objective method to classify foot posture was used to minimise any additional clinician bias in subject selection. A proportion of children who were initially assumed to have PFF were classified as TDF and vice versa (Fig. 1), which shows that some bias has been removed. Even if there remains some selection bias, the aim of this study was not to describe the epidemiology of PFF, but to demonstrate that a proportion of affected children have significantly impaired HRQOL compared to healthy controls. This impairment may be equivalent to or worse than acutely or chronically unwell children.

In conclusion, even though parents may overestimate the severity of their child’s impairment, children with PFF do have significantly impaired HRQOL when compared to TDF children. This is particularly evident with respect to physical functioning and confirms the belief that PFF cannot always be regarded as just a benign normal variant. The diagnosis of PFF alone, however, is not enough to guide clinical management, and careful consideration should be given to the child’s symptom profile and health related quality of life.

## Electronic supplementary material

Below is the link to the electronic supplementary material.

Supplementary material 1 (DOCX 20 kb)
