# Supplementary material for: Health-related quality of life in children with flexible flatfeet: a cross-sectional study
Source: J Child Orthop. 2014 Nov 8;8(6):489–96. doi: 10.1007/s11832-014-0621-0 (PMC4252274; doi:10.1007/s11832-014-0621-0)
Supplement: Supplementary file 1 — Supplementary material 1 (DOCX 20 kb) [file 11832_2014_621_MOESM1_ESM.docx]

Supplementary:- Foot Posture Classification Technique

A number of different measures for foot classification were calculated for each foot of each subject. To obtain these measures each child underwent three dimensional motion analysis, anthropometric measurement, dynamic pedobarography and simulated weightbearing MRI. Below is an outline of how these measures were obtained and the nature of the classification technique.

Anthropometric measures

A number of anthropometric measures of the weight-bearing foot were taken using calipers. These included the absolute foot length (FL) and the truncated foot length (TFL) measured as the foot length with exclusion of the toes. The arch height (AH) was calculated as the vertical distance of the highest point of the dorsum of the foot halfway along the FL. The arch height index (AHI) was calculated as the AH divided by the TFL.

Three Dimensional Motion Analysis

Retroreflective markers were attached to specific anatomical locations on the pelvis and lower limbs of the participant. These locations corresponded to the standard ‘Plug in gait’ model (PiG-Vicon Motion Systems, Oxford UK), as well as the ‘Oxford Foot Model’ (OFM), a clinically tested and validated multi-segment foot model (insert ref). An additional marker was attached to the navicular tuberosity in the standing position. The three-dimensional position of these markers was tracked using 12 infra-red cameras with a sampling rate of 100 Hz (Vicon MX, Vicon, Oxford UK). Data was collected for each child during a standing static trial.

Navicular height was defined as the vertical component of the navicular marker position from the standing static trial. To obtain normalized navicular height (NNH), navicular height was divided by the measured TFL.

Resting calcaneal stance position (RCSP) was obtained from the hindfoot/tibia angle in the coronal plane as calculated from the OFM in the standing static trial.

Dynamic pedobarography

Each child underwent dynamic pedobarography with the Novel EMED-M pressure plate system (Novel, Munich Germany). A three-step technique was used to capture normal dynamic walking data. Five trials for each foot were obtained and the final trial was defined as a representative trial. From this trial, using the EMED-M software, the Arch Index (AI) was calculated as the ratio of the midfoot and truncated foot contact areas as described in Cavanagh, P.R. and M.M. Rodgers, The arch index: a useful measure from footprints. Journal of Biomechanics, 1987. 20(5): p. 547-551.

Simulated weightbearing Magnetic Resonance Imaging (MRI)

Children underwent MRI using the Philips Achieva 3.0 Tesla MRI scanner (Philips medical systems, Da Best, Netherlands).. Subjects underwent three scout images and then a sagittal T1 weighted scan with slice thickness of 2.5 mm. The sagittal plane was orientated according to the long axis of the foot.

The child was positioned on a custom-built rig which was loaded the feet to simulate weight bearing. Each child was loaded with 10 kg to simulate partial weightbearing. MRI images were subsequently uploaded onto MIMICS image processing software (Materialise, Belgium). From the sagittal images calcaneal inclination angle (CIA) was measured as the angle between the plantar aspect of the foot and the line drawn along the under surface of the os calcis at its most inferior projection. The lateral talar first metatarsal angle (T1MA) was also calculated from the sagittal images. This was measured as the angle between the long axis of the talus and the long axis of the first metatarsal.

Classification method.

Table 1 summarises the measured indices considered for foot classification via cluster analysis. This technique aims to group a set of objects in such a way that objects in same group are more similar to each other than those in other groups. It requires that variable used to cluster subjects should not be too highly correlated. As such, all variables for each foot were cross correlated in SPSS (v18, IBM) using Pearson’s R. From the correlation matrix a list of variables were obtained such that no one variable had a correlation coefficient with another of R= ≥0.6. This lead to the exclusion of T1MA and NNH, as both had a correlation of R= ≥0.6 with AHI.

| Foot measures |
| --- |
| Normalized navicular height (NNH) |
| Arch Index (AI) |
| Arch Height Index (AHI) |
| Calcaneal inclination angle (CIA)  Talar 1^st^ metatarsal angle (T1MA) |
| Resting calcaneal stance position (RCSP) |

Table 1. Table summarising foot indices measured for foot posture classification.

The values for each foot of the remaining four variables were analysed with a two-step cluster analysis. The number of groups was not specified and a hierarchical algorithm was selected. The analysis split the feet into two groups. Table 2 summarises the characteristics of the groups for all indices measured, including those excluded from the cluster analysis.

|  | All Feet | | Group 1 (TDF) | | Group 2 (PFF) | |
| --- | --- | --- | --- | --- | --- | --- |
| Variable | Median | SD | Mean | SD | Mean | SD |
| AHI | 0.30 | 0.03 | 0.32 | 0.03 | 0.28 | 0.02 |
| AI | 0.19 | 0.09 | 0.15 | 0.07 | 0.26 | 0.08 |
| CIA(^o^) | 18.03 | 4.54 | 20.12 | 3.20 | 14.33 | 4.19 |
| RCSP(^o^) | -5.78 | 5.26 | -3.84 | 3.73 | -9.19 | 5.84 |
| T1MA | 7.21 | 7.60 | 3.20 | 4.48 | 13.83 | 7.08 |
| NNH | 0.14 | 0.04 | 0.15 | 0.03 | 0.11 | 0.03 |

Table 2. Summary of characteristics of cluster analysis groups for all foot measurement indices

Based on the cluster analysis results, group 1 feet were classified as TDF and group 2 feet as PFF.

The initial foot classification was method was used on 40 subjects. A regression equation was constructed from these results to use as a classification tool for all future subjects. This was used for the remaining 55 subjects.

The equation is as follows:-

$$Group membership=\left( AHI*-72.06 \right)+\left( AI*14.77 \right)+\left( CIA*-0.51 \right)+\left( RCSP*-0.28 \right)+25.45$$

From the results of the equation the probability of group membership can be obtained. If the probability was <0.5 then the foot was classified as PFF and if the value was ≥0.5 then the foot was classified as TDF. In cases where subject’s feet were on the classification border, such that one foot was classified as PFF and the other as TDF, the subject was classified as have PFF. This occurred in 9 individuals. All these individuals were clinically deemed as having PFF, thus this approach seemed reasonable.
